# Association of Parental Environmental Exposures and Supplementation Intake with Risk of Nonsyndromic Orofacial Clefts: A Case-Control Study in Heilongjiang Province, China

**DOI:** 10.3390/nu7095328

**Published:** 2015-08-27

**Authors:** Yanru Hao, Subao Tian, Xiaohui Jiao, Na Mi, Bing Zhang, Tao Song, Le An, Xudong Zheng, Deshu Zhuang

**Affiliations:** 1 Department of Oral Maxillofacial Surgery, The First Affiliated Hospital, Harbin Medical University, 122 Youzheng Street, Nangang District, Harbin 150000, China; E-Mails: honorastar@163.com (Y.H.); mina_doctor@163.com (N.M.); zhangbing_doctor@163.com (B.Z.); zhangxuel@ems.hrbmu.edu.cn (T.S.); anle_doctor@163.com (L.A.); 2 Department of Stomatology, Harbin Children’s Hospital, 57 Youyi Road, Daoli District, Harbin 150000, China; E-Mail: tiansubao_doctor@163.com; 3 Department of Medical Administration, The Second Affiliated Hospital, Harbin Medical University, 246 Xuefu Road, Nangang District, Harbin 150000, China; E-Mail: zhengxudong_doctor@163.com; 4 Department of Stomatology, The Fourth Affiliated Hospital, Harbin Medical University, 37 Yiyuan Street, Nangang District, Harbin 150000, China; E-Mail: stevenchuang88@126.com

**Keywords:** nonsyndromic orofacial clefts, environmental exposures, passive smoking, toxic occupational exposure, multivitamin use, folic acid

## Abstract

The aim of present study was to check the possible association of potential parental environmental exposures and maternal supplementation intake with the risk of nonsyndromic orofacial clefting (NSOC). A retrospective study comprised 499 cases and 480 controls was conducted in Heilongjiang Province. Chi-square analysis and unconditional multiple logistic regression were used in the study. The results showed that maternal history of fever and the common cold without fever (OR_CL/P_ = 3.11 and 5.56, 95%CI: 1.67–5.82 and 2.96–10.47, OR_CPO_ = 3.31 and 8.23, 95%CI: 1.58–6.94 and 4.08–16.95), paternal smoking and alcohol consumption (OR_CL/P_ = 2.15 and 5.04, 95%CI: 1.37–3.38 and 3.00–8.46, OR_CPO_ = 1.82 and 4.40, 95%CI: 1.06–3.13 and 2.50–7.74), maternal exposure to organic solvents, heavy metals, or pesticides (OR_CL/P_ = 6.07, 5.67 and 5.97, 95%CI: 1.49–24.76, 1.34–24.09 and 2.10–16.98, OR_CPO_ = 10.65, 7.28 and 3.48, 95%CI: 2.54–44.67, 1.41–37.63 and 1.06–11.46) and multivitamin use during the preconception period (OR_CL/P_ = 0.06, 95%CI: 0.02–0.23, OR_CPO_ = 0.06, 95%CI: 0.01–0.30) were associated with cleft lip or without cleft palate (CL/P) and cleft palate only (CPO). Maternal history of skin disease and negative life events (OR_CL/P_ = 12.07 and 1.67, 95%CI: 1.81–80.05 and 1.95–2.67) were associated with CL/P. Some potential parental hazardous exposures during the periconception period and maternal use of multivitamins during the preconception period were associated with risk of NSOC.

## 1. Introduction

Orofacial clefting is among the most common of all human congenital birth defects, with an incidence of 1/500 to 1/1000 births worldwide [1]. Numerous studies have demonstrated that the incidence is highest among Asians, followed by Caucasians, and lowest in people of African descent [2]. The ethnic Chinese population has a relatively high incidence of 1.62 per 1000 live births [3]. The majority of clefts, almost 70%, are regarded as nonsyndromic [4]. More than 300 syndromes have been described in which an orofacial cleft is part of the disorder.

Nonsyndromic orofacial clefts (NSOC) are thought to have a multifactorial etiology, with a significant genetic component that likely interacts with a number of environmental factors. Based on epidemiological and embryological data, cleft lip and/or palate and cleft palate are considered distinct forms of orofacial clefting [5]. Food intake, metabolic, hormonal, lifestyle, and genetic factors determine the nutritional status of the mother and could thereby affect organogenesis and growth of the conceptus [6]. Derangements in these molecular processes caused by polymorphisms in developmental genes and/or environmental exposures are suggested to cause nonsyndromic orofacial clefts [7].

Many maternal exposures during first trimester have been reported to be associated with NSOC, including use of anticonvulsants or retinoids [8,9], hazardous habits such as smoking [8,9,10,11] and regular alcohol consumption [9], residential proximity to sites of increased chemical pollution [12], intake of supplementation, and first-trimester fever-producing illnesses including influenza [13,14]. In addition, an association between supplementation containing multivitamins and folic acid and the occurrence of CL/P has been noted [15,16,17]. However, there are some discrepancies in the findings derived from different studies.

Heilongjiang is traditional industrial base in northern China where the major industry is focused upon coal, petroleum, machinery, and food. Also as a large agriculture province, pesticides are commonly used in Heilongjiang. Environmental exposures including occupational exposures, lifestyle factors and consumption of supplementations that were hypothesized to contribute to the risk of oral clefts in this region. Therefore, a hospital-based case-control study was conducted to explore the risk factors of NSOC in Heilongjiang Province, China.

## 2. Material and Methods

### 2.1. Study Design and Participant Selection

We performed a retrospective hospital-based case-control study that involved 499 cases and 480 controls in Heilongjiang Province of China during the period from 2009 to 2014. Cases were recruited from three participating sites in the Smile Train Project: The Affiliated Stomatology Hospital of Harbin Medical University, the Second Affiliated Hospital of Harbin Medical University, and Harbin Children’s Hospital, where most patients with orofacial clefts in Heilongjiang Province and the surrounding areas attend clinics. Cases were patients diagnosed as cleft lip, cleft lip and palate, or cleft palate. Infants with clefts of known origin (amniotic bands, fetal alcohol syndrome, Mendelian inherited disorders, or chromosomal anomalies) were excluded.

Cases were divided into two groups: patients with cleft palate only (CPO group) and cleft lip with or without cleft palate (CL/P group). (Cleft lip and cleft lip with cleft palate share a common development process.) We also randomly selected 480 unrelated healthy controls from the same hospitals after rigorous screening and infants were eligible to serve as a control only if they had no congenital malformations. All infants were under the age of three years when the study was conducted and were residents in Heilongjiang Province. Cases and controls were matched on age and location.

### 2.2. Data Collection

The study protocol was reviewed and approved by the Institutional Ethics Review Board of Harbin Medical University. The informed consent of all participants was obtained before the study began.

We extracted information of case and control mothers from interviewer-administered questionnaires. The interview was administered by a trained interviewer and addressed exposures from one month before conception through the end of the first trimester. The content of the questionnaire mainly comprised sociodemographic characteristics such as maternal gestational age, gender of infant, parental education, annual household income, gravidity, maternal body mass index (BMI), history of negative reproduction parental exposures such as maternal history of illnesses, use of medication, lifestyle behaviors such as smoking, alcohol consumption, negative life events, possible toxic occupational exposures, and use of supplements. Maternal gestational age was considered as a continuous measure and gravidity of the mother was classified as one, two, and three-or-more children. Education was categorized as primary or junior high school, high school or technical school, and university or higher. Annual household income was divided into three levels: Less than 10,000 yuan, 10,000–30,000 yuan, more than 30,000 yuan. Negative reproduction included history of induced abortion, spontaneous abortion, and stillbirth.

In the questionnaires the mothers were asked whether they had a history of fever, common cold without fever or skin disease in the first trimester during pregnancy and then what medication they used to treat them. Parents were considered smokers or to consume alcohol during the first trimester when any smoking or alcohol consumption was reported. Maternal passive smoking was defined as being exposed to the smoke from more than one cigarette per day at home or in the workplace during the first trimester. The occupational exposure of the maternal interview identified mothers who were employed for at least one month during the periconceptional period, defined as one month preceding the conception through the end of the first trimester. Mothers were considered unexposed if they did not have a job or stopped working during the periconceptional period. For each reported job, mothers were asked about the job title, primary tasks and duties, chemicals and machines handled on the job. Mothers were defined as being exposed to organic solvents when reporting contact to industrial cleaning products (degreasers), paints, printing inks or glues in their jobs. They were considered exposed to heavy metals (cadmium, cobalt or lead) exposure if their jobs involved production of pigments or batteries, galvanization, or recycling of electric tools. Mothers were considered exposed to pesticide if they were engaged in agriculture during the periconceptional period. Mothers were considered to have experienced negative life events if they had either of the following: relationship difficulties, legal/financial problems, violence/crime, illness/injury, or a relative’s death.

Data on supplementation intake were derived from the interview. Women were asked whether they used multivitamins, folic acid supplements, or cod liver oil during the one-month preconceptional period or first trimester.

### 2.3. Statistical Analysis

Chi-square analysis and Fisher’s exact test were used to compare frequencies of demographic characteristics and potential exposures between CL/P group, CPO group and control group, respectively. Then the significant data were included in multivariable logistic regression model for further analysis to compare these groups. Analyses were performed using SAS9.1.3. *p*-values less than or equal to 0.05 were considered to be significant. Statistical correction (False Discovery Rate) was applied for the adjustment of the multiple tests.

## 3. Results

### 3.1. Social-Economic Characteristics of the Participants

Table 1 showed the distribution of selected characteristics among mothers of infants in the case and control groups. The distribution of maternal gestational age between the case and control group was significantly different (*p* < 0.001). Mothers of case group had a higher percentage of young age (under 25) than those of control group. Compared with parents of infants in the control group, parents of those in the case group were more likely to be poorly educated (primary or junior high school) and low income (less than 10,000 yuan per year). The distribution of gravidity, history of negative reproduction, and BMI of mothers were different between case and control group. The majority of infants with CL/P were male (69.1%), and over half of infants with CPO were female (64.2%).

As shown in Table 2, maternal history of fever, the common cold without fever and skin disease during the first trimester were found to be associated with all subtypes of NSOC. Use of antipyretics, analgesic and anti-infectious drugs were found to be significantly associated with all subtypes of NSOC. Maternal topical corticosteroids use was associated with CL/P. Paternal smoking was associated with all subtypes of NSOC, as well as paternal alcohol consumption. Maternal passive smoking was associated with all subtypes of NSOC. Additionally, significant associations were found between maternal negative life events during the first trimester and all subtypes of NSOC. Significantly associations were seen for maternal exposure of organic solvents, heavy metals or pesticides, and all subtypes of NSOC.

**Table 1:** Demographic characteristics of 499 case and 480 control parents.

Characteristics	Control *n* = 480 (%)	*n* = 362 (%)	CL/P OR (95%CI)	*p*-Adjusted	*n* = 137 (%)	CPO OR (95%CI)	*p*-Adjusted
Maternal gestational age				<0.001			<0.001
<20	8 (1.67%)	16 (4.4%)	1.85 (0.77–4.43)		8 (5.8%)	2.79 (1.00–7.75)	
20–24	173 (36.0%)	187 (51.7%)	Ref.		62 (45.3%)	Ref.	
25–29	229 (47.7%)	85 (23.5%)	0.34 (0.25–0.48)		36 (26.3%)	0.44 (0.28–0.69)	
30–34	58 (12.1%)	39 (10.8%)	0.62 (0.39–0.98)		22 (16.1%)	1.06 (0.60–1.87)	
≥35	12 (2.5%)	35 (9.6%)	2.70 (1.36–5.37)		9 (6.5%)	2.09 (0.84–5.21)	
Maternal education				<0.001			<0.001
Primary or junior high school	172 (35.8%)	319 (88.1%)	8.04 (5.42–11.92)		110 (80.3%)	4.70 (2.86–7.72)	
High school or technical school	169 (35.2%)	39 (10.8%)	Ref.		23 (16.8%)	Ref.	
University or higher	139 (29.0%)	4 (1.1%)	0.13 (0.04–0.36)		4 (2.9%)	0.21 (0.07–0.63)	
Paternal education				<0.001			<0.001
Primary or junior high school	161 (33.5%)	315 (87.0%)	9.03 (6.08–13.40)		111 (81.0%)	8.27 (4.63–14.78)	
High school or technical school	180 (37.5%)	39 (10.8%)	Ref.		15 (10.9%)	Ref.	
University or higher	139 (29.0%)	8 (2.2%)	0.27 (0.12–0.59)		11 (8.1%)	0.95 (0.42–2.13)	
Household annual income				<0.001			<0.001
<¥10,000	100 (20.8%)	173 (47.8%)	1.94 (1.39–2.70)		58 (42.3%)	1.90 (1.21–2.98)	
¥10,000–30,000	167 (34.8%)	149 (41.2%)	Ref.		51 (37.2%)	Ref.	
≥¥30,000	213 (44.4%)	40 (11.0%)	0.21 (0.14–0.32)		28 (20.5%)	0.43 (0.26–0.71)	
Gender of infant				<0.001			0.002
Male	245 (51.0%)	250 (69.1%)	Ref.		49 (35.8%)	Ref.	
Female	235 (49.0%)	112 (30.9%)	0.47 (0.35–0.62)		88 (64.2%)	1.87 (1.26–2.77)	
Gravidity				<0.001			<0.001
1	309 (64.4%)	164 (45.3%)	Ref.		59 (43.1%)	Ref.	
2	116 (24.2%)	115 (31.8%)	1.87 (1.36–2.57)		47 (34.3%)	2.12 (1.37–3.29)	
≥3	55 (11.4%)	83 (22.9%)	2.84 (1.93–4.20)		31 (22.6%)	2.95 (1.95–4.97)	
Negative reproductive history ^a^				0.002			<0.001
no	345 (71.9%)	223 (61.6%)	Ref.		74 (54.0%)	Ref.	
yes	135 (28.1%)	139 (38.4%)	1.59 (1.91–2.13)		63 (46.0%)	2.18 (1.47–3.22)	
Body mass index (kg/m^2^)				0.004			0.555
<18	58 (12.1%)	62 (17.1%)	1.64 (1.11–2.42)		23 (16.8%)	1.46 (0.86–2.49)	
18–24	384 (80%)	251 (69.3%)	Ref.		104 (75.9%)	Ref.	
25–29	31 (6.5%)	42 (11.6%)	2.07 (1.27–3.39)		8 (5.8%)	0.95 (0.43–2.14)	
≥30	7 (1.4%)	7 (2.0%)	1.54 (0.53–4.41)		2 (1.5%)	1.06 (0.22–5.15)	

CL/P, cleft lip with or without cleft palate; CPO, cleft palate only; ^a^ history of induced abortion, spontaneous abortion and stillbirth; Statistical significance values are given in bold.

**Table 2:** Maternal health factors, occupational exposures, and parental lifestyle factors of cases and controls during the first trimester.

Factors	Control *n* = 480 (%)	CL/P *n* = 362 (%)	*p*-Adjusted	OR (95%CI)	CPO *n* = 137 (%)	*p*-Adjusted	OR (95%CI)
Maternal illness							
History of fever	46 (9.6%)	79 (21.8%)	<0.001	2.63 (1.78, 3.90)	27 (19.7%)	0.004	2.32 (1.38, 3.89)
Cold without fever	36 (7.5%)	120 (33.1%)	<0.001	6.06 (4.05, 9.09)	48 (35.0%)	<0.001	6.65 (4.08, 10.84)
History of skin disease	3 (0.6%)	15 (4.1%)	0.005	6.87 (1.97, 23.92)	4 (2.9%)	0.050	4.78 (1.06, 21.63)
Medication use							
Antipyretics or analgesic ^a^	13 (2.7%)	34 (9.4%)	<0.001	3.72 (1.93, 7.16)	15 (10.9%)	<0.001	4.42 (2.05, 9.53)
Anti-infectious drugs ^b^	20 (4.2%)	74 (20.4%)	<0.001	5.91 (3.93, 9.90)	30 (21.9%)	<0.001	6.45 (3.53, 11.79)
Topical corticosteroids	2 (0.4%)	12 (3.3%)	0.008	8.19 (1.82, 36.82)	3 (2.2%)	0.092	5.35 (0.88, 32.34)
Lifestyle factors							
Maternal smoking	28 (5.8%)	26 (7.2%)	0.452	1.25 (0.72, 2.17)	9 (6.6%)	0.750	1.14 (0.52, 2.47)
Paternal smoking	197 (41.0%)	218 (60.2%)	<0.001	2.17 (1.65, 2.87)	82 (59.9%)	<0.001	2.14 (1.45, 3.15)
Maternal passive smoking	175 (36.5%)	214 (59.1%)	<0.001	2.52 (1.90, 3.33)	71 (51.8%)	0.002	1.87 (1.28, 2.75)
Maternal alcohol consumption	72 (15.0%)	43 (11.9%)	0.177	0.75 (0.50, 1.12)	26 (19.0%)	0.376	1.28 (0.77, 2.11)
Paternal alcohol consumption	74 (15.4%)	170 (47.0%)	<0.001	4.86 (3.52, 6.70)	66 (48.2%)	<0.001	5.02 (3.31, 7.63)
Maternal negative life events	32 (27.5%)	179 (49.4%)	<0.001	2.56 (1.92, 3.42)	69 (50.4%)	<0.001	2.66 (1.81, 3.90)
Maternal occupational exposure							
Organic solvents	8 (1.7%)	22 (6.1%)	0.002	3.82 (1.68, 8.67)	16 (11.7%)	<0.001	7.80 (3.26, 18.66)
Heavy metals	7 (1.5%)	34 (9.4%)	<0.001	7.00 (3.07, 15.99)	12 (8.8%)	<0.001	6.48 (2.50, 16.81)
Pesticides	5 (1.0%)	73 (20.2%)	<0.001	23.99 (9.58, 60.06)	21 (15.3%)	<0.001	17.20 (6.35, 46.60)

^a^ aspirin, acetaminophen; ^b^ antibiotics, antimycotics, antiviral drugs, antihelminthics; OR, odds ratio; CI, confidence interval; Statistical significance values are given in bold.

As shown in Table 3, any use of folic acid during the preconception period (one month before conception) was associated with CL/P. Intake of multivitamins during the preconception period was associated with all subtypes of NSOC. Any use of cod liver oil during the preconception period was associated with all subtypes of NSOC and during the first trimester was associated with CL/P.

**Table 3:** Maternal supplementation intake during the preconception period or the first trimester in case and control group.

Supplementation	Control *n* = 480 (%)	*n* = 362 (%)	CL/P *p*-Adjusted	OR (95%CI)	*n* = 137 (%)	CPO *p*-Adjusted	OR (95%CI)
Folic acid							
Any use during preconception period ^a^	30 (6.3%)	10 (2.8%)	0.025	0.43 (0.21, 0.88)	6 (4.4%)	0.431	0.69 (0.28, 1.69)
Any use during first trimester	97 (24.9%)	67 (18.5%)	0.590	0.91 (0.64, 1.29)	34 (24.9%)	0.257	1.32 (0.84, 2.07)
Multivitamins							
Any use during preconception period ^a^	106 (28.3%)	4 (1.1%)	<0.001	0.04 (0.01, 0.11)	3 (2.2%)	<0.001	0.08 (0.02, 0.25)
Any use during first trimester	85 (17.7%)	47 (13.0%)	0.070	0.69 (0.47, 1.02)	15 (10.9%)	0.084	0.57 (0.32, 1.03)
Cod liver oil							
Any use during preconception period ^a^	30 (6.3%)	1 (0.3%)	0.002	0.04 (0.01, 0.31)	1 (0.7%)	0.048	0.11 (0.01, 0.82)
Any use during first trimester	21 (4.4%)	2 (0.6%)	0.006	0.12 (0.03, 0.52)	1 (0.7%)	0.099	0.16 (0.02, 1.21)

^a^ preconception period means one month before conception; OR, odds ratio; CI, confidence interval; Statistical significance values are given in bold; CL/P, cleft lip with or without cleft palate; CPO, cleft palate only.

### 3.2. Multivariable Analysis

To determine the factors that contributed most to orofacial cleft risk, multiple stepwise logistic regression analyses were performed. The parameters that appeared to be a significant risk factor for orofacial cleft risk in the chi-square analysis were included in the multivariate model. As shown in Table 4, the results of multivariable analysis suggested that maternal history of fever, common cold without fever, history of skin disease and negative life events, paternal smoking and alcohol consumption, maternal exposure to organic solvents, heavy metals or pesticides and any maternal multivitamin use during the preconception period were associated with risk of CL/P. Maternal history of fever and the common cold without fever, exposure to organic solvents, heavy metals or pesticides, paternal smoking, and alcohol consumption and maternal any multivitamin use during the preconception period were associated with risk of CPO.

**Table 4:** Multivariable logistic regression of case and control group.

Factors	Control *n* = 480 (%)	CL/P *n* = 362 (%)	aOR (95%CI)	CPO *n* = 137 (%)	aOR (95%CI)
History of fever	46 (9.6%)	79 (21.8%)	3.11 (1.67, 5.82)	27 (19.7%)	3.31 (1.58, 6.94)
Common cold without fever	36 (7.5%)	120 (33.1%)	5.56 (2.96, 10.47)	48 (35.0%)	8.23 (4.08, 16.59)
History of skin disease	3 (0.6%)	15 (4.1%)	12.07 (1.81, 80.50)	4 (2.9%)	-
Paternal smoking	197 (41.0%)	218 (60.2%)	2.15 (1.37, 3.38)	82 (59.9%)	1.82 (1.06, 3.13)
Paternal alcohol consumption	74 (15.4%)	170 (47.0%)	5.04 (3.00, 8.46)	66 (48.2%)	4.40 (2.50, 7.74)
Maternal negative life events	32 (27.5%)	179 (49.4%)	1.67 (1.95, 2.67)	69 (50.4%)	-
Organic solvents	8 (1.7%)	22 (6.1%)	6.07 (1.49, 24.76)	16 (11.7%)	10.65 (2.54, 44.67)
Heavy metals	7 (1.5%)	34 (9.4%)	5.67 (1.34, 24.09)	12 (8.8%)	7.28 (1.41, 37.63)
Pesticides	5 (1.0%)	73 (20.2%)	5.97 (2.10, 16.98)	21 (15.3%)	3.48 (1.06, 11.46)
Any multivitamin use during preconception period	106 (28.3%)	4 (1.1%)	0.06 (0.02, 0.23)	3 (2.2%)	0.06 (0.01, 0.30)

OR, odds ratio; CI, confidence interval; aOR adjusted for maternal age, sexual of infants, parental education, annual household income, gravidity, maternal body mass index (BMI) and history of negative reproductive; CL/P, cleft lip with or without cleft palate; CPO, cleft palate only.

## 4. Discussion

Orofacial clefts are among the most common human malformations, and both genetic and environmental etiologies can be involved. This study examined the association of several certain environmental exposures with risk of nonsyndromic orofacial clefts and its two subtypes. 

Several previous studies have implicated maternal fever and the common cold during the first trimester as risk factors for orofacial clefts [10,13,14,18]. In accordance with previous studies, we found that maternal history of fever or the common cold was associated with increased risk of orofacial clefts. A cold is a common source of fever, and fever and colds often occur concomitantly. Hyperthermia has been associated with an increased risk of birth defects in both animals and humans [19,20], particularly for neural tube defects [21,22]. High temperature may result in the arrest of mitotic activity and immediate death of cells in mitosis with threshold elevations (1.5–2.5 °C) and a delayed death of cells probably in the S phase with higher elevations (3.5 °C) [20]. Apoptosis is also impacted by hyperthermia [22]. Thus, we raise the hypothesis that increased risks for orofacial clefts by cold could be due to underlying fever.

Numerous studies have been conducted on the association of maternal smoking and alcohol consumption during pregnancy with having a child with orofacial cleft. Some of the studies showed a positive association [10,11,23], other studies showed no statistically significant association [24]. Unlike some previous studies, we did not observe a significant correlation of maternal smoking and alcohol consumption habits with NSOC. Lack of the correlation could be a statistical bias resulting from the low percentage of mothers who smoke and drink in our study. However, the results suggesting that paternal smoking and alcohol consumption may increase the risk of giving birth to offspring with NSOC. It could be plausible that paternal exposures to hazardous agents may induce a genotoxic mutagenic effect on spermatozoa [16].

Maternal occupational exposure to toxic agents seemed *a priori* to have a more direct effect on NSOC, and was previously found to be associated with an increased risk of NSOC in offspring [16]. Heilongjiang is an industrial base and agricultural province in northern China. Its industry mainly focus on coal, petroleum, machinery, and pesticides in the province. Maternal occupation serves as a proxy for environmental and industrial exposures. Significant relation was found between maternal occupational exposure to certain toxic agents (e.g., organic solvents, heavy metals, and pesticides) and increased risk of NSOC in our study. Experimental data supported this: An increased incidence of oral clefts in offspring was noticed when pregnant mice were exposed to the herbicide 2,4,5-trichlorophenoxyacetic acid (2,4,5-T) [25]. One hypothesis of developmental toxicity and teratogenesis of organic solvents was that these compounds produced oxidative stress (OS) to early embryonic development. Animal experiments suggested that OS causes alterations in gene expression and interferes with normal cellular activity of the neural crest cell population, ultimately leading to brain and facial abnormalities [26].

Several observational studies have examined the association of maternal negative life events with risk of orofacial clefts, however, the results were inconsistent [27,28,29]. Similar with a recent study in China [29], maternal negative life events during pregnancy were found to be associated with increased risk of CL/P in our study. Biological plausibility for an association of negative life events during pregnancy with orofacial clefts has been demonstrated in animal models where administration of corticosteroids—a natural product of the stress response—can induce orofacial clefts [30,31].

We found that preconceptional multivitamin use was associated with decreased risk of NSOC. Because multivitamins contained different vitamins and minerals and the composition and dose of vitamins and minerals in multivitamins vary, it was difficult to identify which component(s) of multivitamins possibly contributed to risk reduction. Folic acid is a likely candidate [32]. Type of multivitamins reported in our study was various and a number of which contained folic acid. The effect of folic acid on orofacial cleft prevention may depend on an optimal dose and critical period [33]. The vast majority of mothers interviewed in our study did not plan pregnancy and, as a result, started multivitamins or folic acid uptake during the 2nd or 3rd month of pregnancy, and therefore only as soon as they found out that they were pregnant. Adequate folic acid levels are reached three months later [10]. Consequently, multivitamins or folic acid treatment was null or inadequate during the first gestational trimester, which is the crucial period for craniofacial development. Our data emphasize the need for information campaigns about prompt onset of multivitamins or folic acid supplementation (at least three months before conception) [34]. Moreover, some evidence was observed for decreased risk of NSOC associated with cod liver oil supplement during the preconception period and first trimester in univariate analysis, though the results were excluded from the multivariable analysis. There was no similar research before. For its nutrients, cod liver oil contained vitamin A, D, and E of which vitamin A was an important regulator of embryogenesis [35].

The strengths of this study included its relatively high participation by study subjects, and its separate analysis of CP and CLP, which are likely to be unique etiologic entities. Nevertheless, our study had some potentially important limitations. First, because the study produced many comparisons, some associations may be the result of chance. Confounding was an essential consideration in the design of the study and analysis. Control for confounding was based on multivariable regression that used a standard set of covariates. However, although we adjusted for several potential confounders, we cannot rule out the possibility of unmeasured confounding factors. Thus, it is still possible that residual confounding or effect modification may have influenced the findings. Second, mothers of children in the case group may recall past events and exposures differently than mothers of children in the control group (recall bias). The birth of a baby with a NSOC is a serious traumatic event for most mothers, who therefore attempt to find a causal explanation such as maternal diseases during pregnancy. A similar phenomenon does not occur after the birth of healthy newborn infants. Third, some factors related to NSOC, such as maternal dietary during pregnancy, were not included in our analysis and may correlate with supplementation intake, thus, precision of risk estimation was reduced in some circumstances. Despite these limitations, the risk factors identified in this study may help other researchers to conduct additional etiological studies and to devise more comprehensive strategies for the prevention of orofacial clefts throughout the world.

## 5. Conclusions

In sum, we found evidence of plausible associations between certain environmental risk exposures of the mother and the father with risk of clefts in their offspring. A number of these associations have also been reported in previous studies. These results deserve scrutiny for specific exposures that may be related to orofacial clefts. Furthermore, there is an urgent need for an intensive worldwide education program for healthcare professionals to focus on the prevention of NSOC.
